# High-Throughput Functional MicroRNAs Profiling by Recombinant AAV-Based MicroRNA Sensor Arrays

**DOI:** 10.1371/journal.pone.0029551

**Published:** 2012-01-05

**Authors:** Wenhong Tian, Xiaoyan Dong, Xuerong Liu, Gang Wang, Zheyue Dong, Wei Shen, Gang Zheng, Jianxin Lu, Jinzhong Chen, Yue Wang, Zhijian Wu, Xiaobing Wu

**Affiliations:** 1 College of Life Science, Jilin University, Changchun, Jilin, China; 2 Beijing FivePlus Molecular Medicine Institute, Beijing, China; 3 State Key Laboratory for Molecular Virology and Genetic Engineering, National Institute for Viral Disease Control and Prevention, Chinese Center for Disease Control and Prevention, Beijing, China; 4 Wenzhou Medical College, Wenzhou, Zhejiang, China; 5 Unit on Ocular Gene Therapy, National Eye Institute, National Institutes of Health, Bethesda, Maryland, United States of America; 6 State Key Laboratory of Genetic Engineering, Institute of Genetics, School of Life Science, Fudan University, Shanghai, China; University of Kansas Medical Center, United States of America

## Abstract

**Background:**

microRNAs (miRNAs) are small and non-coding RNAs which play critical roles in physiological and pathological processes. A number of methods have been established to detect and quantify miRNA expression. However, method for high-throughput miRNA function detection is still lacking.

**Principal Findings:**

We describe an adeno-associated virus (AAV) vector-based microRNA (miRNA) sensor (Asensor) array for high-throughput functional miRNA profiling. Each Asensor contains a *Gaussia* luciferase (Gluc) and a firefly luciferase (Fluc) expression cassette to sense functional miRNA and to serve as an internal control respectively. Using this array, we acquired functional profiles of 115 miRNAs for 12 cell lines and found “functional miRNA signatures” for several specific cell lines. The activities of specific miRNAs including the let-7 family, miR-17-92 cluster, miR-221, and miR-222 in HEK 293 cells were compared with their expression levels determined by quantitative reverse transcriptase polymerase chain reaction (QRT-PCR). We also demonstrate two other practical applications of the array, including a comparison of the miRNA activity between HEK293 and HEK293T cells and the ability to monitor miRNA activity changes in K562 cells treated with 12-*O*-tetradecanoylphorbol-13-acetate (TPA).

**Conclusions/Significance:**

Our approach has potential applications in the identification of cell types, the characterization of biological and pathological processes, and the evaluation of responses to interventions.

## Introduction

MicroRNAs (miRNAs) are a species of non-coding RNAs that are 18-25 nucleotides (nt) in length [1] and regulate the expression of one third of the total genes in human cells through perfect or imperfect base pairing with specific sequences of mRNA [2]. The miRNA target sequences in mRNA are primarily located at the 3′ untranslated regions (UTRs) [1], [3] and, in some cases, in the 5′ UTRs [4] or coding regions [5]. The interaction of miRNAs with their target sequences leads to the modulation of gene expression through decreases in mRNA stability and the repression of mRNA translation [6]. These interactions play a critical role in the processes of cell differentiation and proliferation [7], cell death, including apoptosis [8], and tumorigenesis [9].

A number of methods including Northern blot [10], real time polymerase chain reaction (PCR) [11], microarrays [12] and deep sequencing [13] have been developed to detect and quantify miRNA expression. High-throughput miRNA profiling plays an increasingly important role in the characterization of both biological and pathological processes, and possesses the potential for disease diagnosis and prognosis [14]. However, due to variation in miRNA quality and the stringent requirements for each approach, the results of miRNA profiling using these methods are not always reproducible. In addition, miRNA expression levels do not always reflect the authentic activity of each miRNA [15], [16]. The latter correlates with mature miRNA functions and is often affected by multiple steps along the miRNA pathway, including the miRISC forming efficiency, the binding affinity of miRNA to the target sequences at the 3′UTR, and the inhibition efficiency through miRISC binding [17]. Therefore, functional miRNA profiling, which reflects miRNA activity, may display some advantages over conventional miRNA profiling if achievable.

Ideally, miRNA activity should be determined using its natural or predicated target genes' 3′UTRs. However, each miRNA usually regulates the expression of a few genes and has more than one target 3′UTR. Additionally, alternative splicing or polyadenylation for 3′UTR leads to its length and structure changes in some processes. Therefore, it is often not practical to choose an appropriate 3′UTR to assay the activity of a miRNA. To overcome this problem, the current method for assaying miRNA activity typically involves the use of a miRNA sensor that contains a reporter expression cassette with the perfect complementary sequence of the miRNA locating at its 3′UTR [18], [19]. Using this method, miRNA activity is determined by the comparison of reporter expression levels produced by the miRNA sensor with the ‘control sensor’, which carries the identical reporter cassette lacking a miRNA complementary sequence. As a prerequisite, the miRNA sensor plasmids must be efficiently transduced into cells for reporter gene expression. In many cases, this is difficult, particularly when high-throughput transductions are required. We have recently established an adeno-associated virus (AAV) reverse infection (RI) array method, which makes high-throughput *in vitro* transduction efficient and convenient [20]. In this report, we develop an AAV reverse infection array-based dual-reporter system designated as the miRNA Asensor array, which is able to profile miRNA activity in cultured cells. This method allows for convenient, cost-effective, and high-throughput screening of functional miRNA profiles.

## Results

### Establishment of the miRNA Asensor array

**Figure 1:**
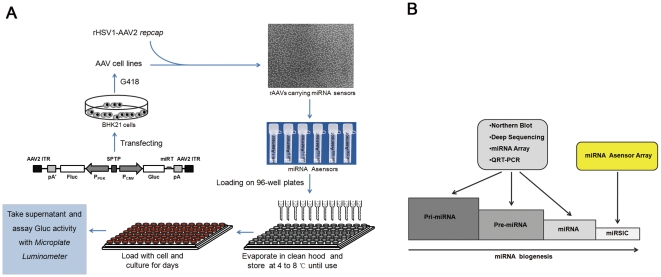
Illustration of the miRNA Asensor array approach. (A) miRNA Asensor array method. SPTP, a synthetic poly(A) signal/transcriptional pause site from the pGL4.14 vector. (B) Specific detection of miRNA activity using the miRNA Asensor array. Methods including Northern blot, miRNA array, deep sequencing, and QRT-PCR are able to detect three forms of miRNA (pri-miRNA, pre-miRNA, and miRNA) during miRNA biogenesis, but can not detect miRSIC (miRNA induced silencing complex), which signals miRNA activity.

The establishment of the miRNA Asensor array is demonstrated in Figure 1A. The miRNA Asensor plasmid was constructed based on an AAV vector plasmid pAAV2neo [20] and contained two independent expression cassettes encoding firefly luciferase (Fluc) and *Gaussia* luciferase (Gluc), respectively (Fig. 1A). The former was used to calibrate the transduction efficiency, while the latter, which included a miRNA perfect complementary target sequence in the 3′UTR of Gluc, was used to monitor miRNA activity. A synthetic poly(A) signal/transcriptional pause site was inserted between the two expression cassettes and reduced the effects of spurious transcription on the Fluc reporter gene expression. Different miRNA Asensor plasmids were constructed by inserting one copy of the corresponding miRNA target sequence into the 3′UTR of Gluc. They were then packaged into recombinant AAVs termed miRNA Asensors (Asensor_miRNA_). The Asensor lacking the miRNA target sequence was termed Asensor_control_. The Asensors were quantified, loaded into 96-well cell culture plates in triplicate, allowed to dry overnight by evaporation in an air clean hood, and stored at 2–8°C until use. In contrast to other miRNA detecting methods, the Asensor array was designed to detect the activity of miRISC (Fig. 1B).

To investigate the relationship between the levels of Asensor loaded per well, and the expression levels of the Fluc and Gluc it generates, serial two-fold dilutions ranging from 5.00×10^8^ to 1.56×10^7^ viral genomes (vg) per well of Asensor_control_ were made. BHK21 cells were added at a density of 1×10^4^ cells per well and cultured for 48 h. Fluc and Gluc activities were then measured separately. The results showed that the expression of Fluc and Gluc exponentially correlated with the levels of loaded Asensor virus (Fig. S1A, B), indicating that the loaded Asensors were not saturated for reporter gene expression within the given range. We selected 2.5×10^8^ vg of Asensor per well as the loading level.

To determine the appropriate cell numbers, BHK21 cells ranging from 3125 to 25000 per well were loaded on the miRNA Asensor array containing 31 miRNA Asensors. The activity of each miRNA was represented by the ratio of Gluc activity of the Asensor_control_ to Asensor_miRNA_, which was defined as the inhibiting fold (IF). The IFs of the miRNAs increased as the number of cells increased from 3125 to 6250 cells per well, but decreased as the cell number further increased from 6250 to 25000 cells per well (Fig. S2A), suggesting that miRNA activity is affected by cell density. However, within the range of the cell numbers tested, the relative activity among each individual miRNA kept constant (Fig. S2B). In addition, the activities of most miRNAs (28 out of 31) did not show significant changes when different cell numbers were applied (Table S1). These results indicate that this approach for functional miRNA profiling is valid within a broad window. We selected 1×10^4^ cells per well as the cell density for the assay thereafter.

To determine the optimal time point for miRNA activity assays, 1×10^4^ HEK293 cells were loaded into each well of the Asensor array containing 31 miRNA Asensors. miRNA activity was then assayed at various time points. Our results showed that the IF for each miRNA Asensor gradually increased and reached its peak value at 48 h, and decreased thereafter as the cells became over confluent (Fig. S2C). However, the proportions of each IF to total IFs in the miRNA Asensor array plate remained almost unchanged (Fig. S2D). For cell lines with a slow growth rate, including BJ and Vero cells, a longer time of culture (4 to 5 days) was required for the IF values to reach their peak (data not shown).

### Presentation of miRNA activity by the relative inhibiting fold

Although equal amounts of each miRNA Asensor were loaded into each well of the 96-well plate, the Fluc activity reflecting the transduction efficiency remained variable among different Asensors due to fluctuations in the titre for each Asensor. To solve this problem, the transduction coefficient (TC) was used to calibrate the miRNA activity obtained by each Asensor. TC calculation for each Asensor performed was as follows.

From Supplementary Figure S1C, without consideration of the miRNA repression of Gluc activity, the relationship between Fluc activity (F) and Gluc activity (G) could be approximated by
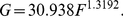
(1)Then the TC value, which is the ratio of G of Asensor_miRNA_ (G_miRNA_) to that of Asensor_control_ (G_control_) when no miRNA repression occurs, could be computed as follows,
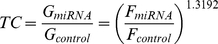
(2)where F_control_ and F_miRNA_ represent the Fluc activity of Asensor_control_ and Asensor_miRNA_, respectively. Since the same batch of Asensor preparation was loaded onto 96-well plates with equal volumes using a 96-well transfer system (Rainin's Liquidator 96, Rainin), the transduction coefficient of each Asensor was constant throughout the experiment. Therefore, the activity of miRNA in a cell line, represented by the relative inhibiting fold (RIF), was calculated as
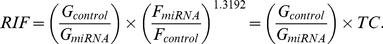
(3)In this study, a total of 115 miRNA Asensors were included in our array plates, and the transduction coefficient for each Asensor on the array in BHK21 cells is shown in Table S2.

### Comparison of miRNA activity and expression levels

**Figure 2:**
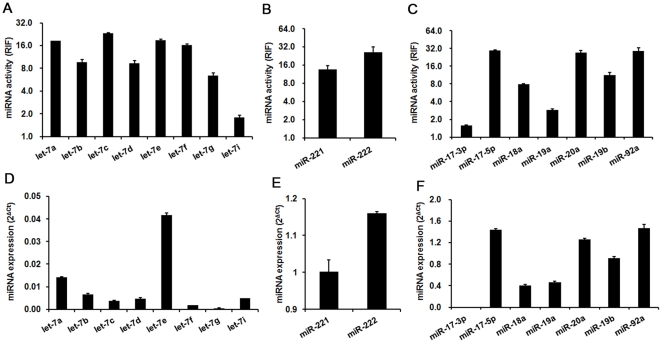
Comparison of miRNA activities with expression levels in HEK293 cells. (A, D) The activities of members of the let-7 family were not consistent with their expression levels. (B, E) miR-221 and miR222 activities were consistent with their expression levels. (C, F) The consistency of miRNA activity with expression levels was complex in the miR-17-92 cluster. For miR-18a, the miRNA activity was not consistent with its expression level. For miR-17-3p, miR-17-5p, miR-19a, miR-19b, miR-20a and miR-92a, the miRNA activities were consistent with their expression levels. 2^ΔCt^ was used to indicate miRNA expression. ΔCt = Ct_U6_−Ct_miRNA_. RIF, relative inhibiting fold. Error bars correspond to mean±SD (n = 3).

To validate the miRNA Asensor array, we compared the activities of miRNAs with their expression levels in HEK293 cells. For members of the let-7 family, it was found that some miRNAs (e.g. let-7a and let-7e) with high expression levels displayed high activity, suggesting the consistency of miRNA activity with expression level (Fig. 2A, D). However, some miRNAs (e.g. let-7c and let-7f) displayed relatively high activity but their expression levels were low (Fig. 2A, D). This result is not surprising, given that each sensor can be recognized by other miRNAs in the family, which share a common seed sequence, and the activity acquired by each sensor reflects the comprehensive effect of the interaction of the sensor with all family members. We further compared the activity and expression levels of members of miRNA clusters, which were divided into two categories. The first category contained several miRNA families (e.g., miR-17-92), whilst the second did not contain miRNA families (e.g., miR-221/222). Our results show that miRNA activity was consistent with expression levels for miR-221/222 (Fig. 2B, E), but not for miR-17-92, which consisted of miR-17-3p and miR-92a, and five other members from two families (one consisted of miR-19a and miR-19b, and the other consisted of miR-17-5p, miR-18a, and miR-20a) (Fig. 2C, F). Taken together, these results indicate that our method can efficiently sense miRNA activity. Furthermore, the miRNA activity results provide information that is complementary to the miRNA expression levels, which will be useful for functional studies of miRNA families.

### miRNAs activity profiles for 12 cell lines

**Figure 3:**
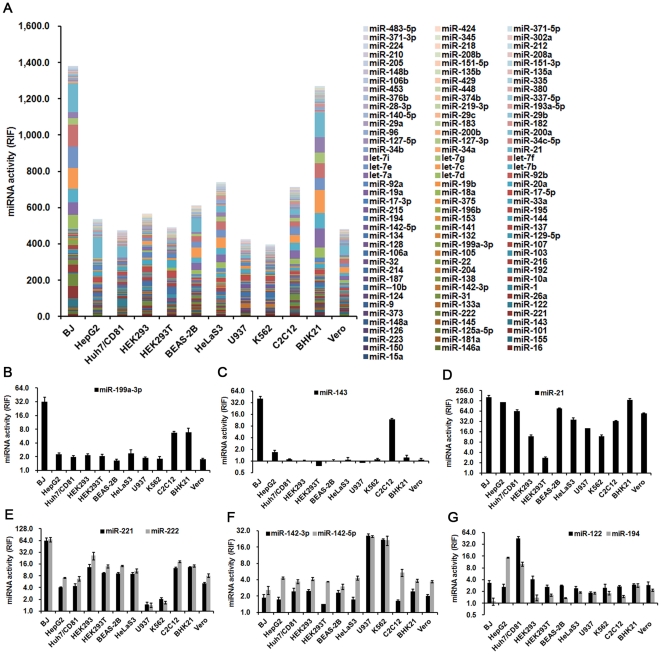
miRNA activity profiles for the twelve cell lines. (A) Activities of 115 miRNAs in 12 cell lines were detected. (B–G) Comparison of specific miRNA activities in 12 cell lines. miR-199a-3p activity was specifically high in BJ, C2C12, and BHK21 cells (B). miR-143 activity was specifically high in BJ and C2C12 cells (C). miR-21 activity was high in all of the cell lines excluding HEK293T (D). miR-221/222 cluster activities were high in all cell lines excluding U937 and K562 (E). miR-142-3p and miR-142-5p activities were specifically high in K562 and U937 cell lines (F). miR-122 activity was specifically high in Huh7/CD81, while miR-194 activity was high in both Huh7/CD81 and HepG2 cells (G). RIF, relative inhibiting fold. Error bars correspond to mean±SD (n = 3).

The RIFs of 115 miRNAs for 12 cell lines were determined using our miRNA Asensor array. Stacked data columns in Figure 3A show the miRNA activity profiles of these cell lines. The total RIF value of the 115 miRNAs for BJ cells was the highest among all 12 cell lines, and much lower for HepG2, Huh7/CD81, K562, U937, and HEK293T cells (Fig. 3A). These results suggested that the total functional activities of the miRNAs were higher in normal tissue derived cells (e.g., BJ and BHK21 cells) and lower in cancer derived cells (e.g., K562 and U937 cells), which is consistent with previous findings [21], [22].

An analysis of the profiles revealed several characteristic miRNAs with the potential to be used as markers for a “functional miRNA signature” of the cell lines. The activities of these miRNAs were either high in all cell lines or uniquely high or low in some cell lines. These miRNAs included miR-199a-3p, miR-143, miR-21, miR-122, miR-194, miR-142-3p and miR-142-5p, and miR-221 and miR-222 (Fig. 3B–G). Among them, the activity of the liver-specific miR-122 [23]–[25] was found to be uniquely high in Huh7/CD81, but absent in HepG2 cells (Fig. 3G). The miR-194 activity was high in both Huh7/CD81 and HepG2 cells, and low or absent in the other tested cell lines (Fig. 3G), suggesting that this miRNA is liver-specific. This result is consistent with previous reports showing that miR-194 is a marker of hepatic epithelial cells [26]. The activities of miR-142-3p and miR-142-5p were specifically high in U937 and K562 cells, consistent with the results from a hematopoietic lineage in a previous study [27]. We found that the activities of miR-221 and miR-222 were high in BJ cells (a human foreskin fibroblast cell line) with RIF values of 63.76±10.33 and 68.04±8.83, respectively, while they were almost absent in U937 (RIF values of 1.47±0.22 and 1.41±0.17, respectively) and K562 cells (RIF values of 2.06±0.17 and 1.64±0.13, respectively) (Fig. 2E). Additionally, miR-143 and miR-199a-3p activities were high in BJ cells (RIF values of 39.80±5.58 and 32.10±8.83, respectively), followed by C2C12 cells (a mouse myoblast cell line; RIF values of 11.79±0.61 and 6.63±0.49, respectively). In contrast, activities were almost absent in other cell lines (Fig. 2B, C), suggesting these miRNAs as probable markers for fibroblast or myoblast cells.

### miRNA activities of two closely related cell lines

**Figure 4:**
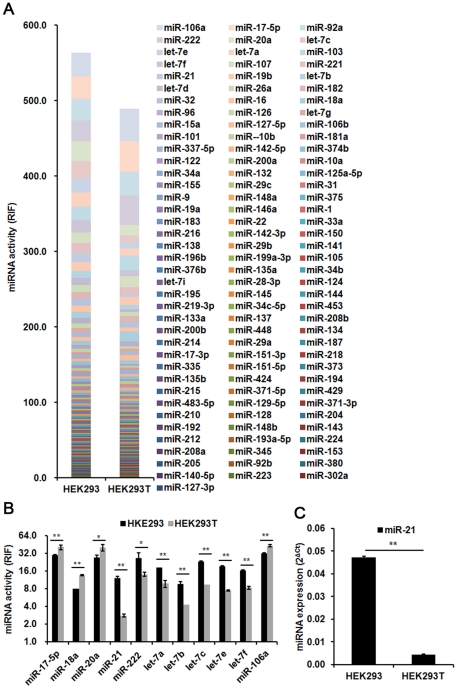
Comparison of miRNA activity profiles between HEK293 and HEK293T cells. (A) miRNA activity profiles for HEK293 and HEK293T were established using the miRNA Asensor arrays. (B) Several miRNAs were selected and their activities compared. Compared to HEK293T cells, miR-17-5p, miR-18a, miR-20a, and miR-106a activities were lower in HEK293 cells, while miR-21, miR-222, let-7a, let-7b, let-7c, let-7e, and let-7f were higher. (C) miR-21 expression level was detected by QRT-PCR. The miR-21 activity was consistent with its expression level in the two cell lines. 2^ΔCt^ was used to indicate the miRNA expression levels. ΔCt = Ct_U6_−Ct_miRNA_. RIF, relative inhibiting fold. Error bars correspond to mean±SD (n = 3). Student T test was used for statistical anylasis. *indicates *P* values<0.05 and ** *P* values<0.01.

HEK293T are HEK293-derived cells that stably express the large T-antigen of the SV40 virus. However, the two closely related cell lines display different miRNA activity profiles (Fig. 4A), indicating the role of the large T-antigen on miRNA activity. miRNAs with RIF values that differed by more than 5.0 between these two cell lines were selected and further analyzed (Fig. 4B). We found that the activity of miR-17-5p, miR-18a, miR-20a (members of the miR17-92 cluster) and miR-106a, which have oncogenic potential [28], [29], were higher in HEK293T cells compared with HEK293 cells (Fig. 4B). In contrast, the activity of let-7a, let-7b, let-7c, let-7e, let-7f (members of the let-7 family) and miR-222, which are known as tumour suppressors [30], [31], were lower in HEK293T compared with HEK293 cells (Fig. 4B). These results suggest that HEK293T cells are more “tumour-like” than HEK293, which may result from the transformation effect of the SV40 T-antigen.

miR-21 is overexpressed in many tumour-derived cell lines, and is believed to promote proliferation through apoptosis inhibition [32]. Interestingly, the activity of miR-21 was lower in HEK293T cell as compared to HEK293 cells (Fig. 4B). To confirm our results, we tested the expression level of miR-21 by quantitative reverse transcriptase (QRT)-PCR (Ambion) (Fig. 4C). The activity of miR-21 was consistent with its expression level for the two cell lines, although they did not correlate proportionally.

### Scanning changes in miRNA activities in response to cell differentiation agents

**Figure 5:**
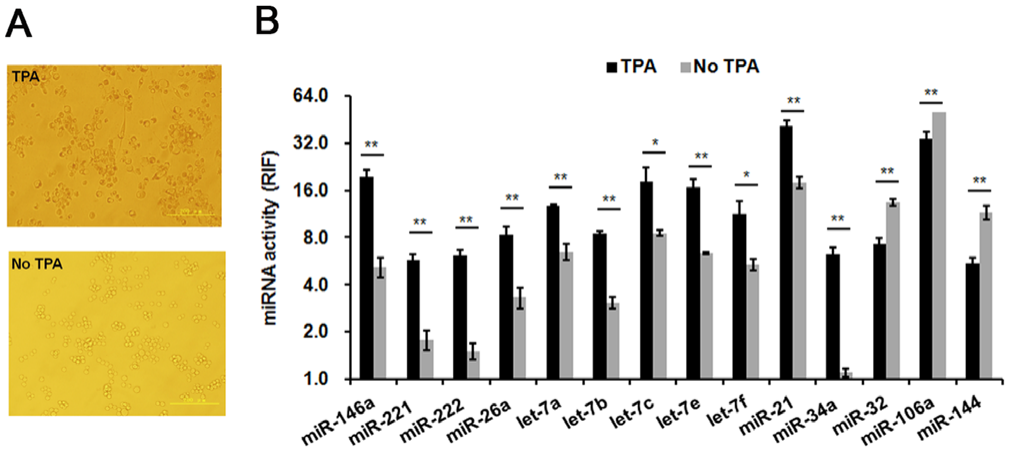
Changes in miRNA activity induced by TPA in K562 cells. (A) Morphological changes in K562 cells induced by TPA. K562 cells were cultured in DMEM (10% FBS) containing 16 nM TPA. Twenty-four hours later, the morphology of K562 cells was altered. (B) Comparison of miRNA activity induced by TPA versus untreated K562 cells. The miRNA activity profiles with or without TPA were detected using miRNA Asensor arrays and miRNA activities with obvious changes are shown. Error bars correspond to mean±SD (n = 3). Student T test was used for statistical anylasis. * *P* values<0.05; ** *P* values<0.01.

To determine the changes in miRNA activity profiles of a cell line during cellular differentiation, we compared 12-*O*-tetradecanoylphorbol-13-acetate (TPA)-treated and untreated K562 cells using our miRNA Asensor array. The miRNA activity profiles did not show significant changes within the first two days (data not shown), most likely due to the fact that TPA-induced differentiation is a gradual process and Gluc must accumulated to high enough levels in each well before differences are demonstrated. However, obvious morphological changes in K562 cells were evident during this period (Fig. 5A). Refreshing the culture medium and re-accumulating Gluc for an additional 24 h made it possible to identify significant changes in miRNA activity (Fig. 5B). We found that miR-34a, miR-221, and miR-222, whose activities were almost absent in untreated cells, significantly increased following TPA treatment (Fig. 5B), consistent with previous studies [33]. Additionally, we found that miR-21, members of let-7 family (let-7a, let-7b, let-7c, let-7e, and let-7f), and miR-146a were upregulated. In contrast, miR-106a, miR-144, and miR-32 were downregulated. These results suggest that the miRNA Asensor array method is well suited for scanning miRNA activity changes during cell differentiation induced by compounds or other agents.

## Discussion

Since its discovery, many approaches for the detection and quantification of miRNA expression have been developed, including Northern blot [10], microarray [12], stem-loop RT-PCR [11], and deep sequencing [13]. All of these methods require RNA extraction, which is troublesome, time-consuming, and leads to bias. Using Northern blot analysis, pri-miRNA, pre-miRNA, and mature miRNA can be distinguished, but the sensitivity of the assay is relatively low. Stem-loop RT-PCR can detect the copy number of mature miRNAs with high sensitivity, but specific primers are required and it is thus difficult to perform in a high-throughput manner. Microarrays are suitable for the high-throughput detection of many miRNAs, but it cannot distinguish pri-miRNA, pre-miRNA, and mature miRNA, and the results are often not reproducible due to variations in miRNA quality. Importantly, the results from these methods cannot reflect miRNA activity that directly involves a post-transcriptional regulation of gene expression. Therefore, a simple, fast, and reliable method for high-throughput functional miRNA profiling is required and will complement existing protocols.

Here, we established a method for high-throughput functional miRNA profiling mediated by miRNA perfect complementary target, nominated AAV vector-based miRNA sensor array. Several features of our AAV vector-based miRNA sensor array make it well suited for performing these tasks. AAV maintains its infectivity in dry conditions [20], making it simple to store and transport the array. miRNA activity from a broad range of cell types can be tested using this array due to its broad cell tropism [34], [35]. The miRNA activity of the cell lines is reflected by the relative inhibiting fold of Gluc expression, which is easily measured [36], [37]. In addition, the procedure avoids the tedious process of RNA preparation. To normalize the transduction by different Asensors on the plates, a transduction coefficient (TC) was determined for each Asensor, which was kept constant in the same batch of Asensor arrays and therefore served as a parameter for quality control. In conventional miRNA profiling protocols, the variability of the results often comes from the quality variation of miRNA. In our current protocol, the major sources of variation were the cell numbers loaded on the array and the time point of assay performance, all of which are easy to control. In addition, our results have shown that the functional miRNA profiles of each cell line were not significantly affected by cell numbers and testing time points, making the assay flexible to use and the results more reproducible. Importantly, the Asensor array is more cost-effective when compared with other miRNA profiling methods. The typical yield of an AAV-microRNA sensor using our AAV production system is between 5×10^11^ to 10^12^ vector genomes per roller bottle, which is sufficient for more than 1000 assays.

The activities of the majority of miRNAs tested by this assay were consistent with their quantities as assessed by QRT-PCR. However, for miRNAs in the same family, such as members of let-7 family, the results of the activity assay were not consistent with their expression levels. This is because the miRNA Asensor mediated by perfect complementary target could “sense” the activity of other members of the same family, and the activity was a comprehensive consequence not only influenced by the corresponding miRNA itself, but also by other members of the same family. Therefore, our method cannot accurately detect specific member activity in miRNA family. Contrarily, the results of our method accurately reflect the status of a specific miRNA target sequence, providing useful information that complements the miRNA expression levels obtained by conventional methods.

Some interesting phenomena were observed when comparing RIF values of an individual miRNA among cell lines. For example, the miR-21 activity was commonly high with an RIF≥10 in all 12 cell lines with the exception of HEK293T. Our recent results also show that the activity of miR-21 is extremely high in mouse hepatocytes *in vivo* (unpublished data). These data argue that miR-21 is an “oncomiR” or a tumour marker, as suggested by earlier reports [38], [39]. Therefore, the role of miR-21 in the cell cycle requires re-evaluation. An additional interesting finding is that the activities of miR-143 and miR-199a-3p are high in human foreskin fibroblast BJ and mouse myoblast C2C12 cells. Previous reports have shown that miR-143 and miR-199a-3p are downregulated in colon cancers [40] and hepatocellular carcinomas (HCCs) [41] respectively, when compared to their adjacent normal tissues. As both fibroblasts and myoblasts are important components of stromal cells [42] and arise from a common population of stromal stem cells [43], it will be critical to identify whether these miRNA expression changes are from authentic tumour and liver cells, or the stromal cells adjacent to those tissues. Although no conclusions can be drawn from the limited data generated from the cultured cell lines, the above results provide important clues that may serve as a starting point to investigate the roles of miRNAs on tissue specific pathways.

In this study, functional miRNA profiles of a total of 12 different cell lines were established using the miRNA Asensor array. As a larger number of cell lines are tested, the functional miRNA signature of each cell line can be stored as a library, which will be invaluable in the identification of unknown cell types, in addition to the investigation of cellular differentiation status. This array is particularly useful when comparing the miRNA activities of two closely related cell lines, such as the same cell line with different passages, cells before and after virus infection or gene transfection, or drug treatment. In this study, we performed two experiments that demonstrate the use of the miRNA Asensor array method. One example is the comparison between HEK293 and HEK293T. The other example is the comparison between K562 cells and those treated with TPA.

In conclusion, we have described the establishment of a miRNA sensor array and its use in profiling functional miRNA in cultured cells. Theoretically, functional miRNA profiling for tissue samples can also be achieved using this array, provided that primary culturing of the tissue samples is successful. In comparison with conventional miRNA profiling, this method is simple and cost-effective. One major limitation of this method is that it only detects miRNA activities within live cells. Therefore, the detection of miRNA in serum and dead cells cannot be achieved. In addition, our method needs Gluc to be expressed in assay cells, which limits its use for some types of cells that are difficult to be transduced by AAV.

However, as an alternative and supplementary approach for miRNA profiling, the miRNA Asensor array described in this study could have wide applications in biology research in addition to the biotech industry, including the identification of cell lines and monitoring changes in miRNA activities in response to chemical compounds, infections, transfections, and other agents. It can also identify miRNA regulators and uncover the mechanisms underlying miRNA activity regulation.

We are planning to expand the number of miRNA Asensors so that the activity of all discovered miRNAs could be tested. Due to the convenience of our Asensor construction approach, the high efficiency of our AAV vector production system and the simplicity of our AAV vector purification method, this task will not be too difficult to fulfil, which could broadly expand the applications of our Asensor array.

## Materials and Methods

### Plasmid construction

A phosphoglycerate kinase (PGK) promoter was inserted into the pGL4.14 vector (Promega) and the resulting new vector was designated pGL4.14-PGK.Using this vector as a template, a fragment containing a PGK-Luc2-SV40 polyA expression cassette and a synthetic poly(A) signal/transcriptional pause site was amplified by PCR and cloned into pAAV2neo -Gluc [20] to generate the Asensor vector plasmid. This Asensor vector plasmid harboured no miRNA target and was used as control. Sense and antisense miRNAs target sequences were synthesized (Table S3) and annealed. Each miRNA target sequence was inserted into the 3′UTR of Gluc of the miRNA Asensor vector plasmid between BglII and EcoRI sites. The correctness was verified by DNA sequencing. A total of 115 plasmids of different miRNA Asensors were obtained.

### Cell culture

BHK21, HEK293, HEK293T, HepG2, K562, U937, HeLaS3, BEAS-2B, C2C12, Vero, and BJ cell lines were purchased from the ATCC (Manassas, VA). And Huh7/CD81 cell line was kindly provided by Dr. Zhuang Hui, professor of Peking University Health Science Center. All cell lines were maintained as monolayer cultures in Dulbecco's Modified Eagle's Medium (DMEM) containing 10% fetal calf serum (FBS), 100 µg/ml penicillin, and 100 U/ml streptomycin, as recommended by the manufacturer (GIBCO). For the differentiation of K562, cells were treated with 16 nM TPA (Sigma Aldrich).

### miRNA Asensors production

Recombinant AAV vectors carrying miRNA sensors (miRNA Asensors) were produced and purified as previously described [44], [45]. Briefly, each miRNA Asensor plasmid was transfected into BHK21 cells using Lipofectamine 2000 (Invitrogen). Asensor cell lines were obtained by culturing with G418 (800 µg/ml) for 15 d. To obtain Asensors, these cell lines were amplified in rolling bottles followed by infection with the recombinant herpes simplex virus carrying a rep-cap gene from AAV2 (rHSV-repcap). About 36 h later, cells were collected and the purification of Asensor was carried out using the method we previously published [45]. The titre of each purified Asensor was determined by dot blotting using a digoxin-labelled cytomegalovirus (CMV) promoter fragment as the probe.

### Preparation of miRNA Asensor arrays

A total of 115 miRNA Asensors and one Asensor_control_ were included in one set of miRNA Asensor arrays. The Asensors were orderly loaded onto 96-well cell culture plates in triplicate using the Liquidator 96 Manual Benchtop Pipetting System (Rainin) with 2.5×10^8^ virus genomes (vg)/20 µl/well. The array was allowed to evaporate in the laminar flow tissue culture hood and stored at 2–8°C until use. Multiple sets of miRNA Asensor array plates were prepared in a batch, making it possible to maintain uniform qualities for each set of the miRNA Asensor array.

### Plating cells onto the miRNA Asensor Array

Cells were digested with 0.25% trypsin (Invitrogen) to create a single-cell suspension. An equal volume of the suspension (200 µl/well) was applied to each well of the miRNA Asensor array plates. The cells were cultured for 48 h at 37°C in an incubator with 5% CO_2_. To obtain optimum results, cells with lower growth rates or a lower transduction efficiency for AAV2 vectors were cultured for 4 to 5 d prior to testing.

### Assays of Fluc and Gluc activity

The Gluc and Fluc assay kits were purchased from New England Biolabs and Promega, respectively. Cells in the 96-well plate were spun down and a 20-µl aliquot of the cell-free medium in each well was taken for Gluc activity assays. Substrate solution (50 µl per well) of Gluc was added with an auto-injector. For Fluc activity, cells were lysed with 100 µl lysis buffer and 20 µl of cell lysate/well was loaded onto 96-well white plates. Substrate solution (50 µl per well) of Fluc was also added with an auto-injector. Both Fluc and Gluc expression were then tested using a luminometer (GloMax®-96, Promega).

### Calculation of transduction coefficients and relative inhibiting folds

BHK21 cells (1×10^4^/well) were added to the miRNA Asensor array plates and cultured for 48 h. Fluc activities of cells in each well were assayed as described above. The transduction coefficients (TC) for each miRNA Asensor were represented by the formula
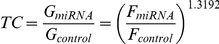
where F_control_ and F_miRNA_ represent the Fluc activity of Asensor_control_ and Asensor_miRNA_, respectively.

The activities of miRNAs in a cell line were represented by the relative inhibiting fold (RIF) calculated as the formula
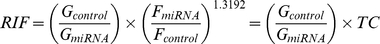
where G_control_ and G_miRNA_ represent the Gluc activity of Asensor_control_ and Asensor_miRNA_, respectively.

### QRT-PCR for miRNA

Total RNA was isolated from 2×10^6^ cells using Trizol reagent (Invitrogen) according to the manufacturer's instruction. RNA concentrations were determined using a BioPhotometer plus (Eppendorf). Each RNA sample (5 ng) was reverse transcribed using a *TaqMan* MicroRNA assay (Ambion) in a 15-µl reaction performed on a thermocycler (Applied Biosystems 9700) for 30 min at 16°C, 30 min at 42°C, 5 min at 85°C, and then held at 4°C. Real-time PCR following reverse transcription (RT) was performed on an Applied Biosystems 7900HT using the same kit. Specifically, 1.33 µl of RT product was used in a 20-µl PCR system. The reactions were incubated in a 96-well plate at 95°C for 10 min, followed by 40 cycles of 95°C for 15 s, and 60°C for 1 min. All reactions were performed in triplicate.

### Statistical Analysis

Student T test was used to analyze the data when miRNA activities were compared between HEK293 and HEK293T cells, or between K562 cells treated with or without TPA. One-way ANOVA was used when miRNA activities were compared among groups with different number of loaded cells.

## Supporting Information

Figure S1 Characterization of control Asensor. (A) The relationship between Fluc activity and the quantity of control Asensor. Control Asensor was serially increased by two-fold and BHK21 cells were loaded. Forty-eight hours later, Fluc activity was tested. (B) The relationship between Gluc activity and quantity of control Asensor. Gluc activity was tested 48 h later. (C) The relationship between Fluc and Gluc activity.
(TIF)

Figure S2 Optimization of the miRNA Asensor array. (A–B) Effect of cell number. BHK21 cells were serially increased by two-fold and loaded into the miRNA Asensor array containing 31 miRNA Asensors. Forty-eight hours later, miRNA activity was detected (A). Proportion of miRNA activity is presented as (B). (C–D) Effect of incubation time. The same amount of HEK293 cells was loaded in the miRNA Asensor containing 31 miRNA Asensors. miRNA activity was detected at different time points (C). Proportion of miRNA activity is presented as (D).
(TIF)

Table S1 Statistical analysis of Supplementary Figure S2A. Activity of specific miRNAs were presented by mean values of IF (Inhibiting fold). One-way ANOVA was used to analyze the data and *P* values were obtained. * indicates *P* values<0.05.
(XLS)

Table S2 Transduction coefficients for miRNA Asensors in BHK21 cells. All of the transduction coefficients (TC) for miRNA Asensors involved in this study were computed based on the formula (F_miRNA_/F_Control_)^1.3182^, in which 1.3182 came from the equation G = 30.983F^1.3182^ deduced from Supplementary Figure S1C and represented the relationship between Fluc and Gluc activity. TC values were represented by mean±SD (n = 3). F_miRNA_, Fluc activity of miRNA Asensor; F_control_, Fluc activity of Asensor_control_; F, Fluc activity; G, Gluc activity.
(XLS)

Table S3 Synthesized oligos for miRNA target. Each miRNA target was prepared by annealing of its forward and reverse sequence. Each forward sequence contains miRNA perfect complementary target with “aatt” at its 5′ end. Each reverse sequence contains a miRNA mature sequence with “gatc” at its 5′ end.
(XLS)
