# Supplementary figures and images for: High-Throughput Functional MicroRNAs Profiling by Recombinant AAV-Based MicroRNA Sensor Arrays
Source: PLoS One. 2012 Jan 5;7(1):e29551. doi: 10.1371/journal.pone.0029551 (PMC3252342; doi:10.1371/journal.pone.0029551)

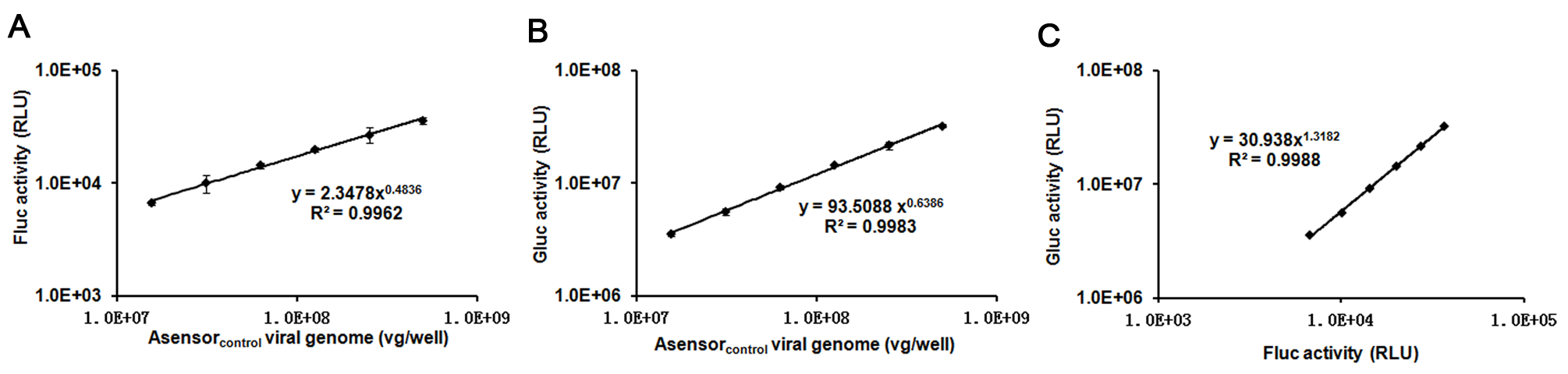

Supplement: Figure S1 — Characterization of control Asensor. (A) The relationship between Fluc activity and the quantity of control Asensor. Control Asensor was serially increased by two-fold and BHK21 cells were loaded. Forty-eight hours later, Fluc activity was tested. (B) The relationship between Gluc activity and quantity of control Asensor. Gluc activity was tested 48 h later. (C) The relationship between Fluc and Gluc activity. (TIF) [file pone.0029551.s001.tif]

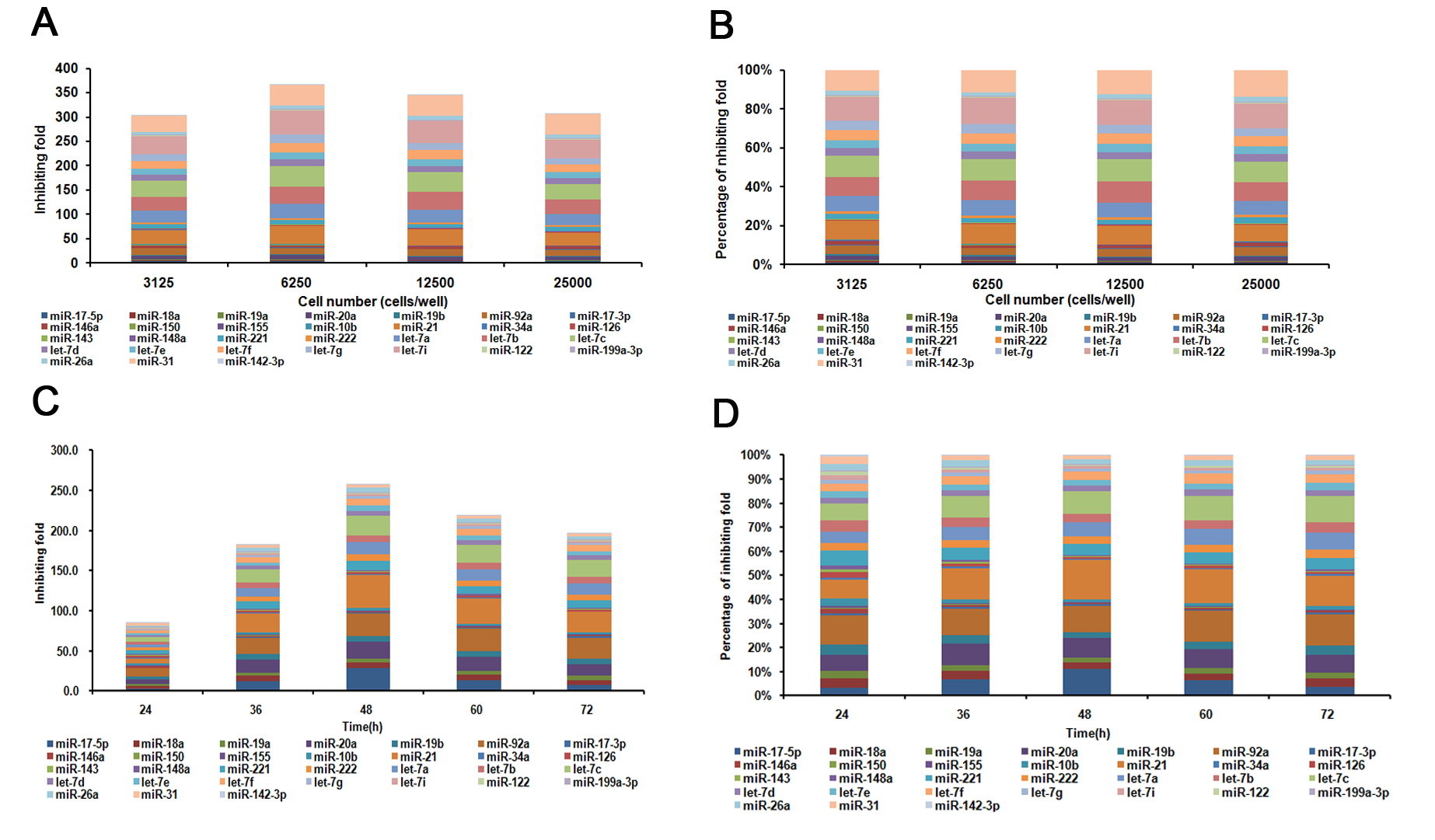

Supplement: Figure S2 — Optimization of the miRNA Asensor array. (A–B) Effect of cell number. BHK21 cells were serially increased by two-fold and loaded into the miRNA Asensor array containing 31 miRNA Asensors. Forty-eight hours later, miRNA activity was detected (A). Proportion of miRNA activity is presented as (B). (C–D) Effect of incubation time. The same amount of HEK293 cells was loaded in the miRNA Asensor containing 31 miRNA Asensors. miRNA activity was detected at different time points (C). Proportion of miRNA activity is presented as (D). (TIF) [file pone.0029551.s002.tif]
